# A Real-Time Learning Analytics Dashboard for Automatic Detection of Online Learners’ Affective States

**DOI:** 10.3390/s23094243

**Published:** 2023-04-24

**Authors:** Mohammad Nehal Hasnine, Ho Tan Nguyen, Thuy Thi Thu Tran, Huyen T. T. Bui, Gökhan Akçapınar, Hiroshi Ueda

**Affiliations:** 1 Research Center for Computing and Multimedia Studies, Hosei University, Tokyo 102-8160, Japan; ho.nguyen.56@hosei.ac.jp (H.T.N.);; 2 Department of Computer Education and Instructional Technology, Hacettepe University, 06230 Ankara, Türkiye

**Keywords:** AI in education, affective states detection, dashboard, learning analytics framework, emotion, lecture video analysis

## Abstract

Students’ affective states describe their engagement, concentration, attitude, motivation, happiness, sadness, frustration, off-task behavior, and confusion level in learning. In online learning, students’ affective states are determinative of the learning quality. However, measuring various affective states and what influences them is exceedingly challenging for the lecturer without having real interaction with the students. Existing studies primarily use self-reported data to understand students’ affective states, while this paper presents a novel learning analytics system called MOEMO (Motion and Emotion) that could measure online learners’ affective states of engagement and concentration using emotion data. Therefore, the novelty of this research is to visualize online learners’ affective states on lecturers’ screens in real-time using an automated emotion detection process. In real-time and offline, the system extracts emotion data by analyzing facial features from the lecture videos captured by the typical built-in web camera of a laptop computer. The system determines online learners’ five types of engagement (“strong engagement”, “high engagement”, “medium engagement”, “low engagement”, and “disengagement”) and two types of concentration levels (“focused” and “distracted”). Furthermore, the dashboard is designed to provide insight into students’ emotional states, the clusters of engaged and disengaged students’, assistance with intervention, create an after-class summary report, and configure the automation parameters to adapt to the study environment.

## 1. Introduction

With the rapid growth of learning technologies, online learning, or virtual education, has become popular. It opened new doors to learners with physical challenges or those who were busy caring for a family that prevented them from learning at school [1]. E-learning has become a frontline approach to supporting education during a crisis such as the COVID–19 pandemic. It is because online learning systems are convenient for students, cost-efficient, flexible ways of learning, scalable, allow for better repetition, and have a higher degree of freedom [2]. With a compelling e-learning system and a highly motivated student, one can achieve great success in a short period of time [2]. On the contrary, online learning environments have several drawbacks, including the fact that they lack the face-to-face interactions that students would receive in traditional classroom settings, and real-time interaction can be frustrating. In addition, it is hard for the lecturer to understand the students’ affective states, such as whether they are feeling confused, motivated to learn, happy with the lesson, taking the lecture with enthusiasm, engaged with the learning materials, or how well the lecture is delivered. 

In online learning, the problem of student disengagement and poor concentration is gaining attention. In the classroom, students’ engagements consist of their behavioral, cognitive, and emotional elements [3]. For example, student academic effort, persistence, attention, concentration, and a lack of conduct problems are associated with behavioral engagement; thoughtfulness and willingness to make the efforts necessary to understand complex ideas and master difficult skills are related to cognitive engagement; and the presence of interest, enthusiasm, the absence of anger, anxiety, and boredom are connected with emotional engagement [3]. It is also evident that students’ engagement is directly proportional to their achievement [4]. Due to poor concentration and a low level of engagement during the lecture, many students fail to achieve the learning goal. This also raises the concern of not receiving a high-quality education. This is because it is challenging for an instructor to keep a close eye on the entire class and regulate teaching. Therefore, for an educational system, it is essential to monitor students’ engagement and concentration frequently, as learning occurs when the students are meaningfully involved in the learning environment. 

To address the above-mentioned issues, this article presents a novel educational application called the MOEMO system with a real-time dashboard. The key contributions of this paper are:

1. To develop an LMS (Learning Management System)-independent learning analytics platform; however, integrable into a chosen e-learning platform such as Moodle, Sakai, and Canvas;
2. The platform could understand students’ motions to detect various affective aspects such as engagement and concentration from the emotional data;
3. To make the platform capable of analyzing the data for valuable educational insights for the lecturers;
4. Visualize the results on the lecturers’ screen in real-time so that they can intervene at the right time while the lecture is in progress;
5. To make the platform suitable for various educational scenarios such as problem-based learning, group discussion, and collaborative learning.

The rest of this paper is organized as follows: In the following Background section (Section 2), we discuss the background of this study, covering why emotion is vital in education, the association between emotion and learning, and learning theories associated with emotion and learning. In Section 3, we provide a literature review covering emotion and learning, emotion detection and ways to detect emotions from logs, sensors, and cameras; and learning analytics dashboards including teacher-centric, student-centric, and human-centric dashboards. Section 4 describes the materials and methods used to build this learning analytics system. Section 5 presents the dashboard and discusses its main components. In Section 6, we provide details on the use case conducted to test the system and understand its usefulness. Finally, the conclusions drawn from this work, along with possible future work lines, are described in Section 7.

## 2. Background

### 2.1. Emotion and Learning: Why Emotions Are Important in Education?

Kop et al. [5] stated that emotions are conceptual entities that arise from brain-to-body-to-environment interactions. Emotions are inherently linked to and influence cognitive skills such as attention, memory, executive function, decision-making, critical thinking, problem-solving, and regulation, all of which play a crucial role in learning [6]. Generally speaking, we have positive emotions and negative emotions that play powerful roles in learning. Positive emotions such as curiosity, passion, interest, wonder, creativity, and joy make our learning experience more desirable and aid in enhancing our focus and attention. These types of emotions enable learners to broaden their perspective and respond effectively to critical situations. On the other hand, negative emotions including sadness, disinterest, disengagement, anxiety, stress, worry, and fear can slow learning processes. These types of emotions negatively impact a learner’s learning experience. While positive emotions help learners stay engaged for longer, negative emotions could be disturbing. Yet negative emotions are not always bad for learning. For example, negative emotions such as confusion can increase students’ interest levels. Additionally, negative emotions are important for learning complex concepts. Therefore, emotions, either positive or negative, are important factors that influence our learning process. Emotions influence our memory, reasoning capability, perception, and logical thinking, and therefore, in education, emotion is essential as it is highly associated with students’ attention [7]. 

### 2.2. Theories Associated with Emotion and Learning

In order to find the relation between emotion and learning, psychologists and neurologists have evaluated many theories. For example, one study [8] evaluated a model in terms of how emotions influence students’ learning and achievement. According to the findings of this study, the influence of emotions can be mediated by several mechanisms with cumulative or contradictory effects on predicting overall effects on performance. The second example is Pekrun’s control-value theory of emotions [9]. The findings of this study indicated that the influence of emotions can be mediated by a few mechanisms with cumulative or contradictory effects for predicting overall effects on performance. 

In addition, affective models are proposed that aim to establish a link between academic emotions and engagement levels. For example, an affective model to identify the level of student engagement depending on their emotions is proposed by Khawlah Altuwairqi [10]. This study conducted a series of experiments to find the relation between engagement levels and their emotions. A study conducted by CR Seal [11] proposed social and emotional development (SED). SED is the integration of theory, emotional intelligence, and competence development applied to educational practice. This study suggests that sustainable enhancement of personal capacity to utilize emotional information, behaviors, and traits could facilitate social outcomes. 

Following these theories, in academics, understanding students’ emotional states is crucial to understanding the learning process. However, despite the importance of recognizing learners’ emotions in e-learning platforms, only a few platforms that recognize learners’ emotions could be found. Hence, new frameworks need to be developed to uncover the unsolved learning problems. 

### 2.3. Measurements of Emotion in E-Learing

Online learning has become the core of research and practices in learning analytics, educational data mining, artificial intelligence in education, intelligent tutoring systems, and educational recommendation systems. The research and practices of learning analytics (LA) are defined as “the measurement, collection, analysis, and reporting of data about learners and their contexts, for purposes of understanding and optimizing learning and the environments in which it occurs” by the Society for Learning Analytics Research (SOLAR). Multimodal learning analytics (MMLA), a subbranch of learning analytics, is focusing research on how critical aspects of the learning process could be revealed using multimodal data such as facial, emotional, gesture, and cognitive data [12]. 

With the help of LA and MMLA, many complex issues, including disengagement in online learning, are explored. Disengagement is regarded as one of the main challenges in online learning. Due to disengagement and poor concentration, many learners fail to achieve their learning goals. To understand more about why disengagement and poor concentration happen, LA and MMLA approaches measure the student’s work context, actions, utterances, facial expressions, body language, and interactions with teachers or fellow students. As of now, many machine learning models, learning analytics systems, and learning analytics dashboards are being developed to regulate learning and teaching. However, measuring emotions and affective states during the class has been a challenge for teachers without LA or MMLA technologies. 

## 3. Literature Review

Emotion and affect are associated with cognitive processes and learning [13]. (Craig et al., 2004); Mayer’s affective cognitive model explains this relationship as follows: the learning episode causes an emotional reaction in the learner that affects cognitive processing during learning and leads to a learning outcome [14]. Studies reveal that different emotional states are associated with task completion and academic success in learning environments. For instance, an intelligent learning environment called AutoTutor [15] has been developed for educational purposes using emotional data. This study found a link between learning and the affective states of confusion, flow, and boredom. In addition, this study also revealed that learning has a positive correlation with confusion and flow but a negative correlation with boredom. In the same line, Whitehill et al. found that students’ engagement levels correlate with their task performance [16]. Sharma et al. also found a correlation between the emotions expressed during the lesson and the concentration level of the students [17]. However, many potentials and aspects of emotional data are yet to come to light. Therefore, researchers are encouraged to collect larger sample sizes. Additionally, more research needs to be conducted about interventions with learning technologies. More precise timing and transitions between emotions are needed to uncover emotion dynamics, and interventions designed to regulate and productively respond to learner emotions are needed [18].

So far, studies conducted on emotion measurement are divided into two groups. The studies in the first group are based on a person’s self-report. The other group of studies relies on biometric measures and other multimodal data sources. Self-report-based studies ask learners to rate how strongly they are experiencing individual emotions, such as anxiety or enjoyment, at a given point in time in the context of a specific activity at hand [14]. For example, the Achievement Emotion Questionnaire, developed by Pekrun et al. [19], is one of the most commonly used emotion surveys used in educational settings. The Geneva Emotion Wheel (GEW) is another self-report instrument used to measure emotion. However, instead of Likert-type questions, a visual card in the form of a wheel is used here, where students can mark their emotional states [20]. The second type of study is non-invasive, and automatic methods based on biometric measurements are used. For example, Sharma et al. proposed a prototype computer vision-based system to measure students’ levels of concentration (e.g., low, medium, and high) in real-time from the expressed facial emotions during a lesson [17]. In another study, Krithika and Lakshmi Priya proposed a system for identifying and monitoring students’ emotions in an e-learning environment by eye and head movements [21]. On the other hand, Whitehill et al. developed a face-recognition-based system that recognizes students’ engagement levels [16].

In the literature, many measurement techniques are employed in learning analytics and psychology to understand why students disengage during class. Although self-report is widely used to measure emotions, there are criticisms of the use of self-reported data. For example, honesty, introspective ability, interpretation, rating scale, validation process, response bias, and sampling bias are the issues for self-reported data. For these reasons, it is clear that there is a need for non-invasive and automatic methods to measure students’ emotional states during their learning processes. Therefore, in this paper, we introduce a new learning analytics framework that could automate the emotion detection process using computer vision and deep learning methods. This learning analytics system uses a computer’s built-in camera function as the source to record lecture videos. These days, having a built-in/web camera is affordable for any student with a laptop or desktop computer; therefore, the system does not rely on any other sensors. The system reads real-time information about the students’ eye movements (eye gazing) and various facial emotions to determine concentration levels. The system reads real-time information about the students’ eye movements (eye gazing) and various facial emotions to determine concentration levels by which an instructor monitors how engaged (or not) the students are. The MOEMO system, in its dashboard, also provides insights on the learners’ emotional states, such as how “neutral”, “fearful”, “disgust”, “angry”, “surprised”, “happy”, and “sad” they are. In real-time, the dashboard shows the instructor the top-3 clusters of engaged and disengaged students based on their engagement score. To regulate learning, it also notifies the instructor that the students have been disengaged for a long period of time so that the instructor can intervene. The use cases of this system could be typical e-learning environments, including collaborative learning, discussion in Zoom’s breakout rooms, peer programming, and lectures related to foreign cultures in cross-cultural language learning scenarios.

## 4. Materials and Methods

### 4.1. The Architecture

**Figure 1:**
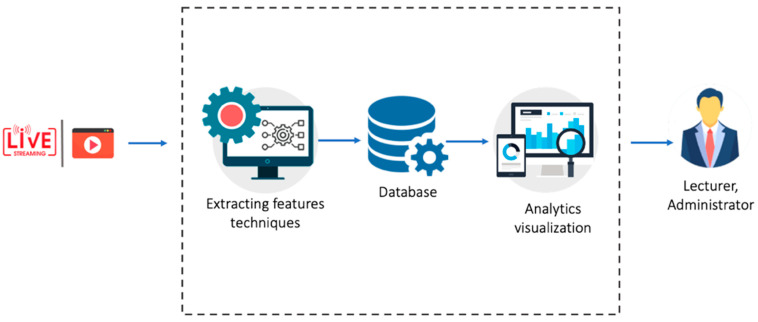
Overall architecture of the platform.

The aim of building this framework is to allow the lecturers to understand their students’ affective states based on their emotions during the lecture. Emotions are captured from students’ facial expressions and eye movements. The key stakeholders of this platform are the lecturers who are delivering lectures using web conferencing tools such as Zoom or WebEx. The MOEMO platform reads raw data (i.e., lecture videos) from web conferencing tools such as Zoom or WebEx to extract students’ emotions. The emotion detection module is the core of the platform, as it provides the system with the capability of collecting, storing, processing, analyzing, and visualizing all the data regarding students’ emotions. The emotion detection module dotes on the system with emotion, engagement, and concentration detection. In addition, this module identifies the clusters of highly engaged and highly disengaged students in a class. Based on this module’s analytics, the lectures know the appropriate intervention time for those students who are highly disengaged and have low concentration. Figure 1 shows the overall architecture of the system. 

**Figure 2:**
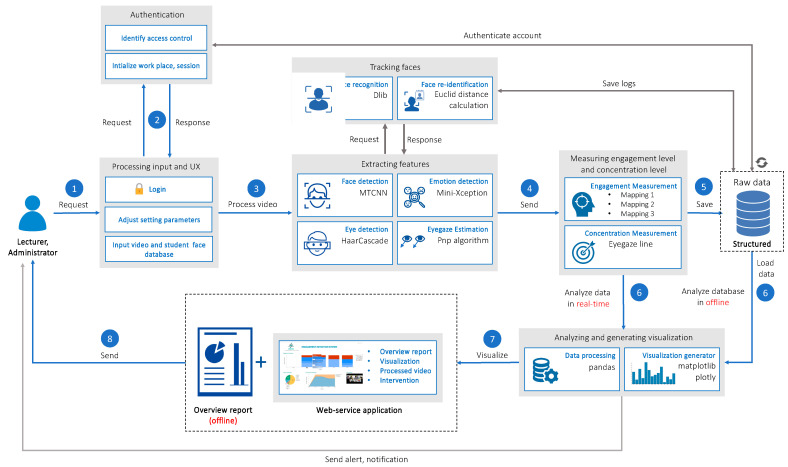
Detailed architecture of the platform.

The technical architecture of the developed platform is shown in Figure 2. First, the system processes the video from the streaming URL or from the recorded video. Then, the system uses computer vision and deep learning techniques to extract emotional information from the entire video. The extracted information, such as face position information, student identification, emotional state, eye gaze, concentration level, and engagement level, is stored in the database. After processing the video, the system proceeds to load data from the database and analyze it to generate analytical data and statistical charts and display them on the web application for lecturers.

In the backend, a general-purpose cross-platform software library called Dlib is used for face recognition tasks. Euclidean distance calculation was performed for the face re-identification task. Face re-identification occurs when a student leaves the web camera and returns after a while. For face detection, MTCNN (Multi-Task Cascaded Convolutional Neural Networks) is used. For various emotion extractions from facial features, the Mini Xception algorithm is used. For eye detection and eye gaze estimation, the HaarCascade and PNP algorithms are used, respectively. For data processing, a Python data analysis library called Pandas is used. To visualize the result on the dashboard, Matplotlib and Plotly’s libraries are used.

### 4.2. Steps

**Figure 3:**
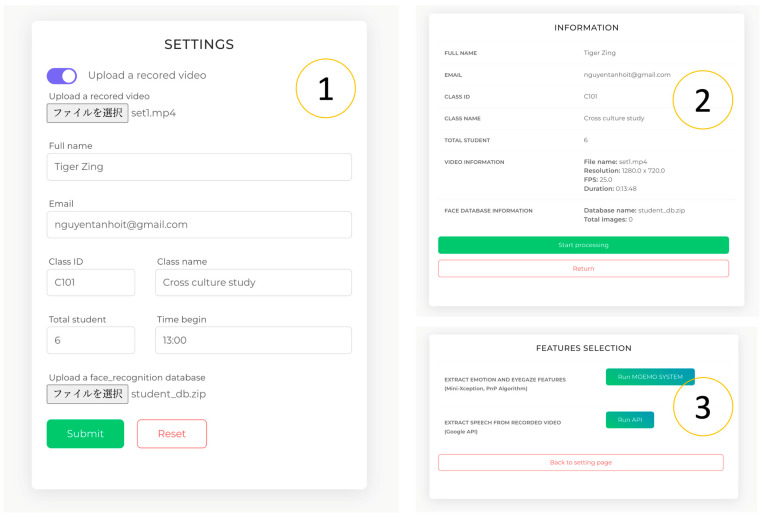
Parameter settings (1), video quality check (2), and customizable feature (3).

As stated earlier, the platform can take recorded video and streaming video from the online class as input. The output of this platform is analytic visualizations. From taking recorded video and streaming video as the input to producing results, there are eight steps involved: 

Step 1. The lecturer needs to input the following information for generating and processing the video. Figure 3 shows the operation of Step 1 in the system.

- First, to upload a recorded video or enter a streaming URL;
- Second, to upload a database for face recognition;
- Third, to customize the setting parameters.

Step 2. Authenticate the account (by the lecturer).

Step 3. Extract behavior features such as the face region, emotion, eye region, and eye gaze line. The methods employed for face detection, face recognition, face re-identification, and emotion detection.

Step 4. Measure the engagement levels and concentration levels. The methods for measuring concentration level and engagement level are articulated in 4.3.5. and 4.3.6., respectively.

Step 5. Save the extracted data to the database.

Step 6. Analyze the data in real-time or analyze the data from the database.

Step 7. Generate the visualization and send it to the web-based service.

Step 8. Visualize the results in the responsive dashboard and send a report to the lecturer.

### 4.3. Methods

#### 4.3.1. Face Detection Method

The Multi-task Cascade Convolutional Neural Network (MTCNN) [22] is employed as the face detection method. This deep learning method is one of the state-of-the-art recent methods that have achieved state-of-the-art results on standard benchmark face detection datasets. The MTCNNs consist of three stages. In the first stage, it produces candidate windows quickly through a shallow convolutional neural network. Then, it refines the windows to reject a large number of non-face windows through a more complex convolutional neural network. Finally, it uses a more powerful convolutional neural network to refine the result and output facial landmark positions. This pre-trained model is trained on a dataset including the face detection dataset and benchmarks FDDB [23], WIDER FACE [24], and the annotated facial landmarks in the wild (AFLW) benchmark [25]. Table 1 shows the datasets used to train the model.

#### 4.3.2. Face Recognition Method

Dlib library is used for face recognition. The Dlib model has achieved an accuracy of 99.38% on the standard benchmarks for face recognition. This accuracy means that, when the Dlib model is asked to correctly identify a pair of faces of the same person, it would detect the right face 99.38% of the time. The model transforms a human face into a 128–dimensional vector space where the images of the same person are near each other and images from different people are far apart. Therefore, performing face recognition by mapping faces to the 128–dimensional vector space and then checking if their Euclidean distance is small enough (using a distance threshold of 0.6) is possible. However, it requires a database that includes all students’ faces.

#### 4.3.3. Face Re-Identification Method

The main idea behind implementing the face re-identification model lies in estimating the small distances (Euclidean distance) from a face position in the current frame compared to the face positions in the previous frame. In short, the pair of similar faces will have the shortest Euclidean distance. The Euclidean distance between two points in Euclidean space is the length of a line segment between the two points. It can be calculated from the Cartesian coordinates of the points using the Pythagorean theorem, therefore occasionally being called the Pythagorean distance. The distance between (q_1,q_2) and (p_1,p_2) is given by:

(1) $$d\left(q,p\right)=\sqrt{{\left(q_{1}-p_{1}\right)}^{2}+{\left(q_{2}-p_{2}\right)}^{2}}$$

The condition is that the movement of the face between the two adjacent frames is tiny. If the movement is significant, we use face recognition to identify all the faces in the current frame. If there is any face that cannot be identified, we set the face as a new face. 

For the experimental purpose, we first saved the face position as a central point (Face_x, Face_y). Then, we calculate the Euclidean distance between the face position in the current frame and all other face positions in the previous frame. The matching face will have the smallest distance. Note that the distance calculation is only suitable in cases where the movement of a face across two frames is very small. For that reason, a threshold is necessary to determine the magnitude of the minimum distance. The concept of a "threshold" in the context of face movement is pertinent to the two factors that influence such movement: the size of the face and the distance of the frame for processing. The purpose of this threshold is to decide when we need to use face recognition (refer to 4.3.2) or face re-identification methods. This threshold will depend on the size of the face and the distance between the two frames. In the real-world scenario, a user is able to adjust the “gap” between two frames for processing in the system. For example, if we obtain 1 frame every 3 seconds to process in a video with 25 fps, the “gap ratio” will be (75-1)/3*25. In short, the large size and the long distance between the two adjacent frames cause significant motion. This means if the gap ratio is bigger than the threshold, we will apply face recognition because the gap between the two frames is too long for us to apply face identification. If the gap ratio is smaller than the threshold, we can apply face identification due to the short gap between the two frames. For example, if we process 1 frame every 3 seconds, it leads to a large motion.

(2) $$threshold=1.5*\max\left(width,height\right)+2*\Delta f$$

where (width, height) is the size of the face and

f is the distance between two frames, with 

f<(3*fps) meaning that distance calculation cannot be used if we process the frame every 3 seconds because of unidentified motions. The face re-identification method saves much more time than the face recognition method. Therefore, we need to use this threshold to save the processing time as much as possible.

#### 4.3.4. Emotion Detection Method

Mini–Xception [26], which is one of the state-of-the-art methods for extracting emotion from facial expressions, was used for this study. The Mini–Xception model has achieved an accuracy of around 95.60% on the FER-2013 dataset [27]. This convolutional neural network (CNN) model has been used to build various real-time systems. However, we faced several challenges related to the performance of the Mini–Xception model that may affect the performance of emotion detection. For example, (i) the detected emotion may not be accurate when a student is speaking without facing the camera, (ii) when a student is doing something off-task, such as playing with a smartphone, and (iii) the orientation of the face may affect the accuracy of emotion detection. At present, the MOEMO system extracts seven types of basic emotions, namely “angry”, “disgust”, “fear”, “happy”, “sad”, “surprise”, and “neutral”. Here, our challenges included when the face is covered by objects, emotion detection under bad light, and when speaking or engaging in off-task behavior.

#### 4.3.5. Concentration Level Measurement Method

**Figure 4:**
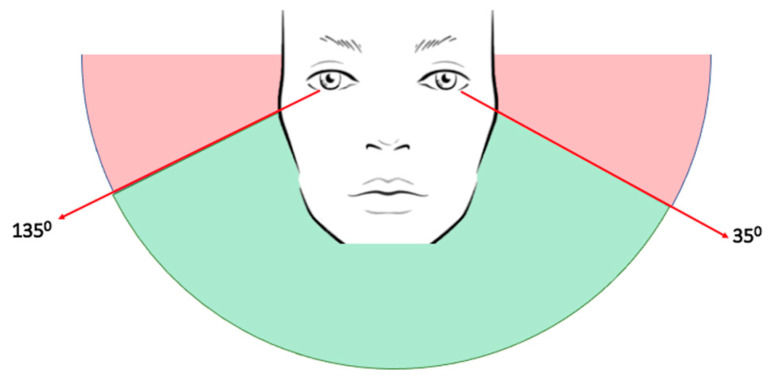
The focused_state and distracted_state detection method for identifying concentration level.

In concentration level measurement, we leveraged eye gaze information to determine the concentration level. In particular, we calculate the angle of the human eye with respect to the camera and the length of the eye gaze line (as shown in Figure 4). We implemented the flowing pseudo-code for determining if the student is looking at the screen or looking away from the screen.

if (degree >= 35 and degree <= 135) and length_eyegaze < length_threshold: return **focused_state** else: return **distracted_state** (3)

We employed the Perspective-n-Point (PnP) algorithm—a widely used technique in computer vision and computer graphics for determining the position and orientation of a three-dimensional (3D) object in a scene based on its 2D projection in an image. Eye detection and feature extraction methods are first applied to the image to obtain the eye_center point, eye_gaze line, and eye_view using the PnP algorithm. Once the eyes are localized, landmarks are extracted using techniques such as the Active Shape Model (ASM) or the Active Appearance Model (AAM), which enable estimation of the geometric center of the eye or the eye-center point. It is worth mentioning that other methods, such as Haar cascades, Viola-Jones, or deep learning-based methods, can be used for this purpose. The eye_gaze line, which denotes the direction of the gaze, is computed by drawing a line from the eye_center point in the direction of the gaze. This is achieved by estimating the rotation and translation parameters of the camera using the PnP algorithm and projecting the eye_center point onto the image plane. The direction of the eye_gaze line is then obtained by calculating the vector connecting the projected eye_center point with the image center. By transforming the eye position into the coordinate system of the camera using the estimated head pose and projecting it onto the image plane using the camera’s intrinsic parameters, it is possible to calculate the eye view. In the above pseudo-code, we use the math functions math.atan2 and math.degrees to calculate the degree between two points: the eye_center point and the eye_view point.

(4) $$degree=math.degrees(math.atan2(P_{eye\_center},P_{eye\_view}))$$

where $P_{eye\_center}$ is the eye center point $\left(x_{eye\_center},y_{eye\_center}\right)$ and $P_{eye\_view}$ is the eye view point $\left(x_{eye\_view},y_{eye\_view}\right)$. 

The eye gaze line is a way of showing where the eye is looking in 3D space. The longer the eye’s gaze line, the farther the eye can see. After conducting many experiments, we have found a threshold for the eye gaze line that can help us determine the quality of someone’s vision, which means the person is looking at the screen or not. To understand how well someone can see between their eye and a computer/laptop, we set a benchmark or a standard (length_threshold). This threshold helps us decide whether their visual range is good or not. After testing the system using various intervals, we set this interval [35°–135°] as an allowed threshold for determining if the student is looking at the screen or looking away from the screen. Furthermore, the length of the eye gaze line is considered when measuring concentration level. The length of the eye gaze line indicates the distance between the eye and the point of view. In short, the shorter the length, the closer the student looks to the camera. In this system, we set the length_threshold as:

#### 4.3.6. Engagement Detection Method using Emotion Data

In this research, students’ engagements are detected using the mapping method described in the related literature [7]. The calculation of engagement detection is based on the concentration index calculation method proposed by Sharma et al. [17]. The mapping method uses simple assumptions and mathematical grounds to identify student engagement from emotional data. 

## 5. The Dashboard

### 5.1. Design of the Dashboard

**Figure 5:**
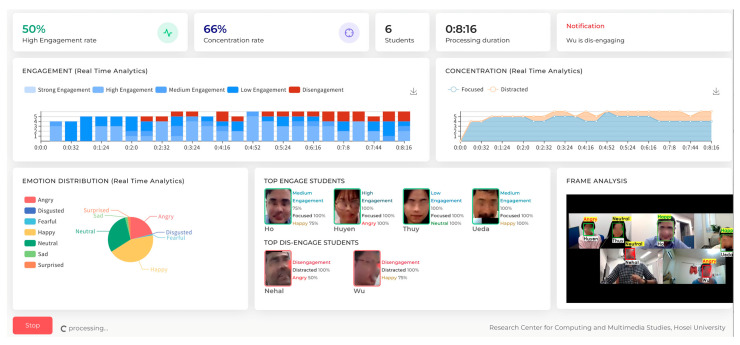
The dashboard.

The dashboard of the MOEMO system provides a detailed insight into the student’s affective states on a lecturer’s screen while the class is in progress (that is, in real–time). The dashboard can generate an after–class report. It is designed in a way that the instructors can interpret the graphs and other visualizations as efficiently as possible. The analytics engine is designed to run in real time and update related tables in the database. Figure 5 shows the dashboard.

### 5.2. Components of the Dashboard

The current version of the dashboard has eleven elements to support instructors in understanding affective states, concentration levels, and engagement levels. Table 2 summarizes the elements, update intervals, and descriptions of the dashboard elements.

#### 5.2.1. Engagement Prediction Panel and Engagement Graph

The dashboard contains an engagement prediction panel where instructors can see the prediction made by the system on their students’ engagement rate. The standardized scores between 0 and 100 are generated from their emotional data. The dashboard also has an engagement graph that visualizes online learners’ five types of engagement, namely “strong engagement”, “high engagement”, “medium engagement”, “low engagement”, and “disengagement”. This way, an instructor could see the overall engagement rate in the classroom. 

#### 5.2.2. Concentration Prediction Panel and Concentration Graph

The dashboard is supported by a concentration prediction panel where instructors can see the prediction made by the system on their students’ concentration rate. As with the engagement rate, the standardized scores between 0 and 100 are generated from students’ emotional data. The concentration graph on the dashboard visualizes two types of concentration, which are “focused” or “distracted”. 

#### 5.2.3. Notification Panel

The notification panel is the intervention system designed to inform instructors about students who have been disengaged for a long time. When a student is disengaged for more than 30 seconds, a notification appears on the notification panel. By looking at the notifications, the instructor can intervene to increase engagement. The time to set intervention notifications could be easily customized. For instance, if a teacher is unhappy with a 30–second intervention, they could select their intervention time.

#### 5.2.4. Cluster Panel

The cluster panel, in real–time, shows the clusters of top-engaged and top–disengaged students. These clusters are generated based on student emotional data. The cluster panel is updated every second.

#### 5.2.5. Emotion Distribution

In the pie chart on the dashboard, the distribution of overall emotions is shown to the instructor. Seven types of emotional states, such as “neutral”, “fearful”, “disgust”, “angry”, “surprised”, “happy”, and “sad”, are shown on the dashboard. By seeing this pie chart, the instructor can quickly understand whether students enjoy the class and how their affective states are at a particular class time.

#### 5.2.6. Frame Analysis

The dashboard contains a video player with frame analysis capability. This video player shows, in real-time, students’ affective states, such as how “neutral”, “fearful”, “disgusted”, “angry”, “surprised”, “happy”, and “sad” they are.

#### 5.2.7. After-Class Report Generation

**Figure 6:**
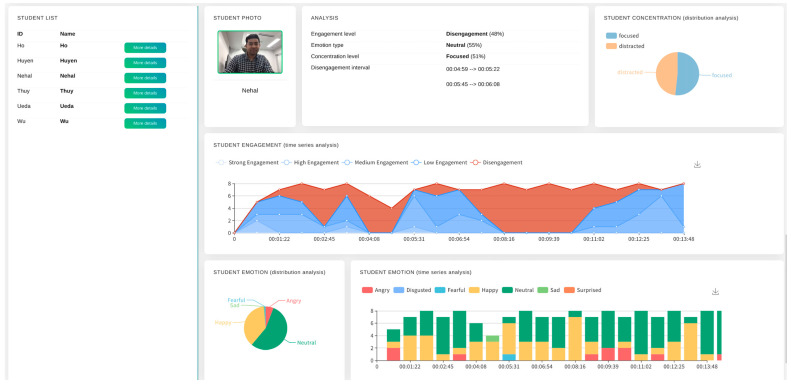
After-class report generation.

The dashboard is supported by an after-class report generation function. Using this function, an instructor could generate a report after the class. This report comprehensively details students’ affective states, concentration, engagement, and intervention time. Figure 6 shows the insight of the after-class report.

## 6. Case Study

In order to test the system and better understand the usefulness of the dashboard, we conducted a case study. We created a class called “*Cultural Exchange on Kanji*” in this case study. The class was formed with one teacher and five students. The students have the cultural backgrounds of Vietnam (N = 3), China (N = 1), and Bangladesh (N = 1). The teacher was native Japanese. Before the case study, we explained the purpose of this case study to the teacher and the students, describing how they allow us to extract their emotional data to visualize their affective states, engagement level, and concentration level. The topic of discussion was “How is Kanji used in your culture?”. The discussion covered the history and revolution of Kanji in China and Vietnam. The class was conducted using the Zoom video conferencing tool. The class was 9 minutes and 9 seconds long. We visualized the result on the teacher’s display during the class. We also generated an after-class report for the teacher. The result of this case study is presented in Figure 5 and Figure 6.

## 7. Conclusions and Future Works

The emotions of online learners can be identified by the patterns in their ability to think, respond, communicate, or behave in an educational context. Human beings are highly influenced by emotion when making decisions and exhibiting behaviors with their surroundings and other aspects of their well-being [28]. In education, emotions are very important. To describe the importance of emotion in learning, Damasio elaborates, “emotions are inseparable from the ideas of reward and punishment, pleasure and pain, approach and withdrawal, personal advantage and disadvantage. In organisms equipped to sense emotions—that is, to have feelings—emotions have an effect on the mind, as they occur, in the here and now. Emotion is devoted to an organism’s survival (quoted) [29]”. 

In online learning, disengagement and low concentration levels are critical issues that teachers, researchers, and educational institutions often face. Concurrently, understanding students’ affective states is challenging in online education. Therefore, a teacher-support tool is essential to monitor students’ engagement and concentration frequently, as learning occurs when the students are meaningfully involved in the learning environment. In this study, we designed and developed a framework to analyze lecture videos to understand students’ affective states, engagement levels, and concentration levels from their facial features. We employed computer vision and deep learning methods to extract emotion from students’ faces. After that, we constructed a database for handling emotional data. In real-time, the analytics engine of the proposed MOEMO system provides insight into engagement prediction, concentration prediction, classroom overview, processing duration, notification of intervention, engagement graph, concentration graph, student list, clusters of engaged and disengaged students, emotion analytics, frame analysis per second, and after-class report generation. The dashboard of the system could be operated in real-time or offline. Therefore, this work’s main experimental contribution was designing and implementing a real-time learning analytics dashboard that could automatically detect online learners’ affective states using deep learning and computer vision techniques.

However, this study has several limitations that will be addressed in future work. The first limitation is a proper evaluation of the system in terms of academic performance, and the accuracy of the data has yet to be determined. To address this limitation, a set of hypotheses was raised: (H1) Being aware of online learners’ affective states helps an instructor guide them to better academic performance; (H2) the dashboard’s quality as a learning analytics tool to monitor online learners’ engagement and concentration; and (H3) the types of engagement (i.e., five types) and concentration (i.e., two types) presented on the dashboard are standardized. Therefore, the extended work would test these hypotheses with the dashboard’s usage data. The second limitation is the validation of the emotional data. To overcome this limitation, we aim to validate the data by collecting feedback from online learners. One considered approach is to validate the data by implementing the Geneva Emotion Wheel (GEW: https://www.unige.ch/cisa/gew/ (accessed on 19 April 2022)). The third limitation is that we could not ensure to what extent the dashboard can provide relevant information about student behaviors so that the lecturers can monitor, observe, and measure students’ engagement, concentration, and intervention time. Therefore, the extended work will undoubtedly explore this.

## Figures and Tables

**Table 1:** Datasets.

No	Name	Description
1	FDDB	Contains the annotations for 5171 faces in a set of 2845 images.
2	WIDER FACE	Consists of 393,703 labeled face bounding boxes in 32,203 images.
3	AFLW	Contains the facial landmarks annotations for 24,386 faces.

**Table 2:** Components of the dashboard.

Dashboard Function	Update Interval	Description
Engagement prediction panel	Realtime	Overall engagement rate (range 0 to 100%)
Concentration prediction panel	Realtime	Overall concentration rate (range 0 to 100%)
Classroom overview	Once (before class)	Number of students in the class
Processing duration	Realtime	Total processing time of the lecture video
Notification panel	Realtime	Intervention
Engagement graph	Realtime	Engagement level in each minute
Concentration graph	Realtime	Concentration details
Cluster panel	Realtime	Top engaged and disengaged students
Emotion distribution	Realtime	Overall emotional rate of the class
Frame analysis	Realtime	Shows the affective states
After class report	Once (after class)	Report provides a detailed insight of the class’s affective states
